# *Porphyromonas gingivalis*-derived lipopolysaccharide confers chemotherapy resistance and migratory ability on oral cancer cells by activating toll-like receptor 4 signaling pathway

**DOI:** 10.1007/s11033-026-11713-1

**Published:** 2026-03-27

**Authors:** Yoshiteru Yamashita, Hideo Shigeishi, Sho Yokoyama, Ryo Uetsuki, Fumie Shiba, Shigehiro Ono, Kouji Ohta, Tomonao Aikawa

**Affiliations:** 1 Department of Oral and Maxillofacial Surgery, Program of Dentistry, Graduate School of Biomedical and Health Sciences, Hiroshima University, 1-2-3 Kasumi, Minami-ku, 734-8553 Hiroshima, Japan; 2 Department of Public Oral Health, Program of Oral Health Sciences, Graduate School of Biomedical and Health Sciences, Hiroshima University, 1-2-3 Kasumi, Minami-ku, 734- 8553 Hiroshima, Japan; 3 Collaborative Research Laboratory of Oral Inflammation Regulation, Graduate School of Biomedical and Health Sciences, Hiroshima University, 1-2-3 Kasumi, Minami-ku, 734-8553 Hiroshima, Japan

**Keywords:** Oral squamous cell carcinoma, Lipopolysaccharide, *Porphyromonas gingivalis*, Chemotherapy resistance

## Abstract

**Background:**

This study examined the *Porphyromonas gingivalis*-derived lipopolysaccharide (*P. g*-LPS)-activated-toll-like receptor 4 (TLR4) pathway and its association with resistance to chemotherapy in oral squamous cell carcinoma (OSCC) cells.

**Methods and results:**

The OSCC cell lines OM-1 and HOC621 were used in this study. Cells were treated with *P. g*-LPS, TAK-242 as a TLR4 inhibitor, SC75741 as a nuclear factor-kappa B (NF-κB) inhibitor, and AH-6809 as a prostaglandin E2 (PGE_2_) receptor antagonist. 5-Fluorouracil (5-FU)-induced cytotoxicity was investigated by measuring lactate dehydrogenase that leaked from damaged cells. 5-FU-induced cytotoxicity was attenuated by *P. g*-LPS in OM-1 cells and HOC621 cells, although this attenuation was relieved by TAK-242, TLR4 siRNA knockdown, or SC75741. *P. g*-LPS induced phosphorylation and nuclear translocation of NF-kB/p65, and enhanced cyclooxygenase 2 (COX2) expression and PGE_2_ production in OM-1 cells. 5-FU-induced cytotoxicity was enhanced by AH-6809 in *P. g*-LPS-treated OM-1 cells. Furthermore, *P. g*-LPS-promoted cell migration was inhibited by TAK-242 or SC75741 in OM-1 cells. This study also involved an additional experiment focusing on CD44^high^ OSCC cells. 5-FU-induced cytotoxicity was attenuated by *P. g*-LPS in amoeboid CD44^high^ OM-1 cells when cultured on laminin-332-coated silicone gel. Additionally, *P. g*-LPS enhanced cofilin-1 dephosphorylation and cell protrusion in amoeboid CD44^high^ OM-1 cells.

**Conclusions:**

*P. g*-LPS is involved in chemotherapy resistance of OM-1 cells by activating the TLR4/NF-kB pathway, which promotes the migratory ability of these cells, and enhancing PGE_2_ autocrine signaling. *P. g*-LPS plays a vital role in OSCC cells’ acquisition of a highly malignant phenotype.

**Supplementary Information:**

The online version contains supplementary material available at 10.1007/s11033-026-11713-1.

## Introduction

Periodontitis is one of the most common inflammatory oral diseases characterized by chronic inflammation of periodontal tissue. Among periodontitis-related bacteria, *Porphyromonas gingivalis* is a Gram-negative anaerobic bacterium associated with severe chronic periodontitis [1]. The importance of *P. gingivalis* in the pathogenesis of periodontitis involves its production of virulent factors such as fimbriae, gingipain, and lipopolysaccharides (LPSs), which facilitate the destruction of periodontal tissues [1]. *P. gingivalis*-derived LPS (*P. g*-LPS) is involved in the destruction of periodontal tissues by modulating the secretion of cytokines such as interleukin (IL)-6, IL-8, and tumor necrosis factor-alpha (TNF-α) in human gingival fibroblasts [2].

LPS also binds to toll-like receptor 4 (TLR4) in host immune cells and promotes an inflammatory response by inducing their secretion of proinflammatory cytokines [3]. In addition, in human cancer cells, LPS enhances cell proliferation, invasion, and resistance to apoptosis by activating TLR4 signaling [4, 5]. However, the impact of *P. g*-LPS on chemotherapy-induced cytotoxicity has not been comprehensively investigated in oral squamous cell carcinoma (OSCC) cells.

LPS activates the TLR4–nuclear factor-kappa B (NF-κB) signaling pathway and triggers a proinflammatory response [6, 7]. Additionally, the TLR4 signaling pathway is involved in the activation of cyclooxygenase 2 (COX2) in human cancer cells [8]. COX2 facilitates the synthesis of prostanoids such as prostaglandin and is implicated in cancer progression by enhancing the immune evasion of cancer cells [9]. Prostaglandin E_2_ (PGE_2_), a product of the COX2 pathway, promotes the evasion of apoptosis in cancer cells by modulating the function of immune cells in the tumor microenvironment [10]. We previously reported that cancer-derived PGE_2_ inhibits 5-Fluorouracil (5-FU)-induced apoptosis via prostaglandin E receptor in OSCC cells [11]. These findings indicate that LPS-induced TLR4 signaling may be involved in chemotherapy resistance in OSCC cells.

This work also involved an additional experiment focusing on CD44. This focus was chosen because CD44^high^ OSCC cells were reported to exhibit cancer stem cell properties such as high tumorigenicity and metastatic potential [12]. Such cells cultured on soft silicone gel displayed an amoeboid round morphology and acquired strong resistance to chemotherapy [13]. Therefore, it is speculated that amoeboid CD44^high^ OSCC cells with cancer stem cell properties contribute to the poor prognosis in OSCC cases. Cofilin-1 has also been shown to play an important role in cell motility by promoting rearrangement of the actin cytoskeleton and the local formation of cell protrusions in amoeboid cells [14, 15]. The activation of cofilin-1 via its dephosphorylation was also shown to be involved in the migratory ability of amoeboid CD44^high^ OSCC cells [13]. However, no reports have described the effects of periodontopathic bacteria on amoeboid CD44^high^ OSCC cells. Therefore, this study also examined the impact of *P. g*-LPS on 5-FU-induced cytotoxicity and cofilin-1 dephosphorylation in amoeboid CD44^high^ OSCC cells.

## Materials and methods

### Cells, cell culture, and treatment

The OM-1 cell line was established from a metastatic lymph node in a case of human tongue squamous cell carcinoma [16], while the HOC621 cell line was established directly from human tongue squamous cell carcinoma [17]. These OSCC cells were cultured in Dulbecco’s Modified Eagle Medium-high glucose (Sigma–Aldrich, St. Louis, MO, USA) supplemented with 10% fetal bovine serum (Biological Industries, Kibbutz Beit-Haemek, Israel) and 1% penicillin/streptomycin (Sigma–Aldrich). Cells were maintained at 37 °C with humidified 5% CO_2_. CD44^high^ OM-1 cells were sorted using anti-human CD44 antibody (BD Pharmingen, San Diego, CA, USA) by fluorescence-activated cell sorting as described in our previous report [18]. CD44^high^ OM-1 cells were cultured on 2.0 kPa silicone gel-coated culture plates (Advanced BioMatrix, Inc., San Diego, CA, USA) coated with 5.0 µg of laminin-332 (Veritas, Tokyo, Japan). Cells were treated with 5.0 µg/ml *P. g*-LPS (InvivoGen, San Diego, CA, USA), 1.0 µg/ml *Escherichia coli*-derived LPS (*E.coli*-LPS) (InvivoGen), 2.0 µM TAK-242 (Selleck Chemicals, Houston, TX, USA) as a TLR4 inhibitor, 2.0 µM SC75741 (MedChemExpress, Monmouth Junction, NJ, USA) as an NF-κB inhibitor, 2.0 µg/ml AH-6809 (Cayman Chemical, Ann Arbor, MI, USA) as a PGE_2_ receptor antagonist, and 1.0 µg/ml PGE_2_ (Tokyo Chemical Industry Co., Ltd., Tokyo, Japan).

### Cytotoxicity assay

Cytotoxicity was determined using Cytotoxicity LDH Assay Kit-WST (Dojindo Laboratories, Tokyo, Japan) by measuring lactate dehydrogenase (LDH) leaked from damaged cells. Cell culture supernatant was collected after 72 h of treatment with 250 µg/ml 5-FU (Wako, Osaka, Japan). Absorbance was measured using the iMark Microplate absorbance reader (Bio-Rad Laboratories, Hercules, CA, USA) at a wavelength of 490 nm.

### Tumor sphere formation assay

Sphere formation assay was conducted to assess the extent to which the tumor cells exhibited the properties of cancer stem cells. Cells were gently resuspended in 3D Tumorsphere Medium XF (PromoCell, Heidelberg, Germany). A total of 1.0 × 10^3^ cells were plated per well in Costar 24-well Ultra-Low Attachment Plates (Corning Life Sciences, Tewksbury, MA, USA). The number of tumor spheres (> 150 μm in diameter) was counted at 10 days of culture.

### Scratch wound healing assay

A scratch wound healing assay was conducted to examine the migratory ability of OM-1 cells. Cells were seeded at a density of 1.0 × 10^5^ cells/well in 12-well culture plates. A scratch was made by scraping the confluent monolayer using a 1.0 ml pipette tip with an outer diameter of approximately 1.8 mm. Photographs were then taken under a phase contrast microscope at 0, 24, and 48 h. Scratch area at each time point was calculated using ImageJ software, with scratch healing being calculated using the following formula: Scratch healing (%) = (scratch area at 0 h − unhealed wound area at 24 h or 48 h) / scratch area at 0 h × 100 (%).

### Invasion assay

CytoSelect 24-well Cell Invasion Assay Kit (Cell Biolabs, Inc., San Diego, CA, USA) was employed to measure cell invasiveness. A serum-free suspension solution containing 1.0 × 10^6^ cells/ml was added to the upper chamber of the kit, after which cell culture medium was added to the lower chamber. After 48 h of incubation, invasive cells on the bottom of the insert membrane were removed using 225 µL of detachment buffer and counted using a Countess Automated Cell Counter (Invitrogen, Waltham, MA, USA).

### siRNA knockdown

For siRNA knockdown, 10 nM Stealth RNAi™ siRNA (Thermo Fisher Scientific, Waltham, MA, USA) and Stealth RNAi™ siRNA Negative Control were transfected using Opti-MEM™ I Reduced Serum Medium (Thermo Fisher Scientific) and Lipofectamine RNAiMAX Transfection reagent (Thermo Fisher Scientific), in accordance with the manufacturer’s instructions. The sequences of siRNA are presented in Table S1.

### Real-time polymerase chain reaction (PCR) analysis

Complementary DNA was synthesized from total RNA using ReverTra Ace qPCR RT Master Mix (Toyobo Life Science, Osaka, Japan). Real-time PCR was conducted with a Thermal Cycler Dice Real Time System III (Takara, Osaka, Japan) using Thunderbird SYBR qPCR Mix (Toyobo Life Science). A previously reported primer set for amplifying DNA was used in this study [19–27]. The sequences of primers employed in this study are summarized in Table S2. The PCR cycling process was 95 °C for 2 min; followed by 40 cycles of 95 °C for 30 s, 58–60 °C for 30 s, and 72 °C for 30 s; and finally 72 °C for 2 min. *GAPDH* was used to normalize the target gene expression.

### Western blotting

Cells were lysed using RIPA lysis buffer and the lysate was centrifuged at 15,000 rpm for 10 min. The supernatant containing total protein was boiled with sample buffer at 95 °C for 3 min. Protein was separated by sodium dodecyl sulfate polyacrylamide gel electrophoresis, followed by electrotransfer onto nitrocellulose membranes. Enhanced chemiluminescence western blotting reagent (Cytiva, Marlborough, MA, USA) and a cooled CCD camera system (LAS-4000; Fujifilm, Tokyo, Japan) were used to detect western blot bands. Antibodies used for western blotting are summarized in Table S3. The intensities of bands in the western blotting were analyzed by ImageJ software, version 1.47 (NIH, Bethesda, MD, USA).

### Immunofluorescence

NF-κB p65 mouse monoclonal antibody (Proteintech) and Alexa Fluor 488 goat anti-mouse antibody were used for NF-κB p65 staining. F-actin staining was performed using Alexa Fluor 568 phalloidin (Thermo Fisher Scientific). Cofilin-1 was stained using anti-Cofilin-1 rabbit monoclonal antibody (Cell Signaling Technology) as a primary antibody and Alexa Fluor^®^ 488 goat anti-rabbit antibody (Thermo Fisher Scientific) as a secondary antibody. Fluoromount-G (Southern Biotech, Birmingham, AL, USA) containing 4′,6-diamidino-2-phenylindole (DAPI) was used to stain the cell nuclei. Fluorescence images were observed using Biorevo BZ-9000 (Keyence).

### Measurement of PGE_2_ concentration

PGE_2_ concentrations in culture medium were measured using Prostaglandin E_2_ ELISA Kit (Cayman Chemical), in accordance with the manufacturer’s instructions. After 48 h of incubation of the cells in culture medium without FBS, the culture medium was collected to determine the PGE_2_ concentration. The absorbance values were measured using the iMark Microplate absorbance reader with a 405 nm filter.

### Statistical analysis

All statistical analyses were conducted using JMP Pro (version 17; SAS Institute Inc., Cary, NC, USA). Comparisons of continuous variables between two independent groups were performed using an unpaired t-test, while those among three or more groups were performed using one-way analysis of variance (ANOVA) with post-hoc Tukey’s honestly significant difference (HSD) test. All results are shown as the mean ± standard deviation. *P*-values of < 0.05 were regarded as statistically significant.

## Results

### *P. g*-LPS inhibited 5-FU-induced cytotoxicity in OM-1 cells and HOC621 cells

**Fig. 1:**
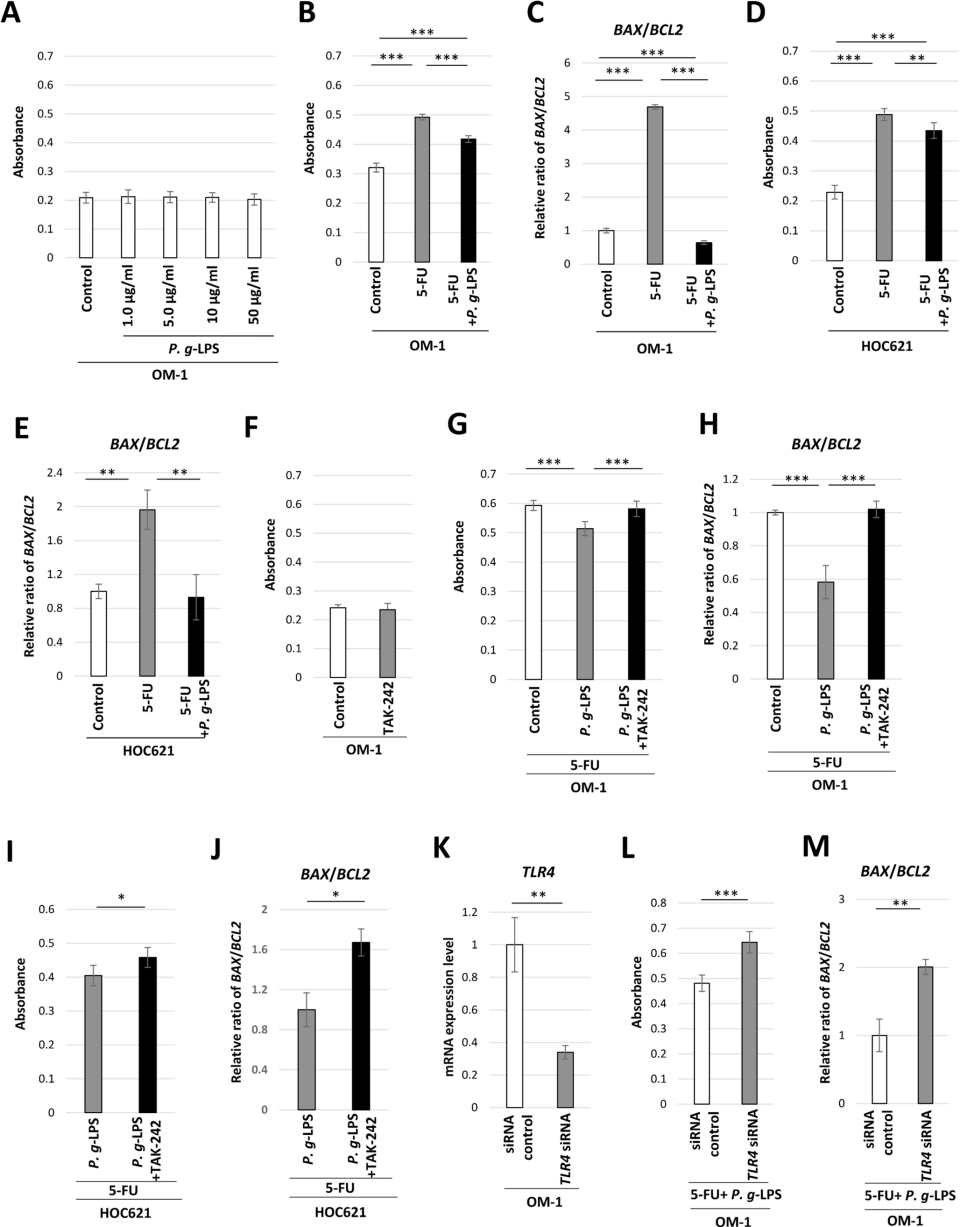
5-FU-induced cytotoxicity in *P. g*-LPS-treated OM-1 cells and HOC621 cells. (**A**) Cytotoxicity assay in the presence of *P. g*-LPS at concentrations of 1.0, 5.0, 10, and 50 µg/ml. (**B**) Cytotoxicity assay in 5-FU + *P. g*-LPS-treated OM-1 cells (***P < 0.001, one-way ANOVA with post-hoc Tukey’s HSD test). (**C**) Relative expression ratio of *BAX*/*BCL2* mRNA in 5-FU + *P. g*-LPS-treated OM-1 cells (**P < 0.01, one-way ANOVA with post-hoc Tukey’s HSD test). (**D**) Cytotoxicity assay in 5-FU + *P. g*-LPS-treated HOC621 cells (**P < 0.01, ***P < 0.001, one-way ANOVA with post-hoc Tukey’s HSD test). (**E**) Relative expression ratio of *BAX*/*BCL2* mRNA in 5-FU + *P. g*-LPS-treated HOC621 cells (**P < 0.01, one-way ANOVA with post-hoc Tukey’s HSD test) (**F**) Cytotoxicity assay in TAK-242-treated OM-1 cells. (**G**) Cytotoxicity assay in *P. g*-LPS + TAK-242-treated OM-1 cells in the presence of 5-FU (***P < 0.01, one-way ANOVA with post-hoc Tukey’s HSD test). (**H**) Relative expression ratio of *BAX*/*BCL2* mRNA in *P. g*-LPS + TAK-242-treated OM-1 cells in the presence of 5-FU (***P < 0.01, one-way ANOVA with post-hoc Tukey’s HSD test). (**I**) Cytotoxicity assay in *P. g*-LPS + TAK-242-treated HOC621 cells in the presence of 5-FU (*P < 0.05, unpaired t-test). (**J**) Relative expression ratio of *BAX*/*BCL2* mRNA in *P. g*-LPS + TAK-242-treated HOC621 cells in the presence of 5-FU (*P < 0.05, unpaired t-test). (**K**) *TLR4* mRNA expression levels in siRNA control OM-1 cells and *TLR4* siRNA knockdown cells (**P < 0.01, unpaired t-test). (**L**) Cytotoxicity assay in siRNA control OM-1 cells and *TLR4* siRNA knockdown cells in the presence of 5-FU + *P. g*-LPS (***P < 0.001, unpaired t-test). (**M**) Relative expression ratio of *BAX*/*BCL2* mRNA in siRNA control OM-1 cells and *TLR4* siRNA knockdown cells in the presence of 5-FU + *P. g*-LPS (**P < 0.01, unpaired t-test)

The cytotoxic effect of *P. g*-LPS on OM-1 cells was first examined in this study. No significant increase of LDH leakage was found in the presence of *P. g*-LPS at concentrations of 1.0, 5.0, 10, and 50 µg/ml compared with the findings in the control (Fig. 1A). *P. g*-LPS at 5.0 µg/ml was employed in the following experiments. 5-FU-induced LDH leakage from damaged cells was inhibited by *P. g-*LPS in OM-1 cells (Fig. 1B). The mRNA expression ratio of *BAX*/*BCL2* was also reduced by *P. g*-LPS in 5-FU-treated OM-1 cells (Fig. 1C). Additionally, the impact of *P. g-*LPS on 5-FU-induced cytotoxicity was examined in HOC621 cells. 5-FU-induced LDH leakage from damaged cells was inhibited by *P. g-*LPS in HOC621 cells (Fig. 1D). The mRNA expression ratio of *BAX*/*BCL2* was reduced by *P. g*-LPS in 5-FU-treated HOC621 cells (Fig. 1E). LDH leakage from damaged cells induced by a lower concentration of 5-FU (25 µg/mL) was also inhibited by *P. g-*LPS in OM-1 cells after 72 h (Fig. S1A). The mRNA expression ratio of *BAX*/*BCL2* was reduced by *P. g*-LPS in OM-1 cells treated with 25 µg/mL 5-FU (Fig. S1B). It is thus suggested that *P. g*-LPS also inhibited cytotoxicity induced by a low concentration of 5-FU in OM-1 cells. Next, the impact of TLR4 inhibition on 5-FU-induced cytotoxicity was examined in OM-1 cells. No significant cytotoxic effect of the TLR4 inhibitor TAK-242 was found in OM-1 cells (Fig. 1F). Meanwhile, the inhibition of LDH leakage by *P. g*-LPS was alleviated by TAK-242 in 5-FU-treated OM-1 cells (Fig. 1G). The mRNA expression ratio of *BAX*/*BCL2* was also elevated by TAK-242 treatment in 5-FU and *P. g*-LPS-treated OM-1 cells (Fig. 1H). Additionally, the attenuation of LDH leakage by *P. g*-LPS was alleviated by TAK-242 in 5-FU-treated HOC621 cells (Fig. 1I). Moreover, the mRNA expression ratio of *BAX*/*BCL2* was elevated by TAK-242 treatment in 5-FU and *P. g*-LPS-treated HOC621 cells (Fig. 1J).

Next, the impact of *TLR4* siRNA-mediated knockdown on 5-FU-induced cytotoxicity was examined in *P. g*-LPS-treated OM-1 cells. *TLR4* mRNA expression was significantly attenuated in OM-1 cells after *TLR4* siRNA knockdown compared with that in siRNA control cells (Fig. 1K). 5-FU-induced LDH leakage was enhanced in *TLR4* siRNA knockdown cells compared with that in siRNA control cells in the presence of *P. g*-LPS (Fig. 1L). The relative mRNA expression ratio of *BAX*/*BCL2* was also elevated in *TLR4* siRNA knockdown cells compared with that in siRNA control cells in the presence of 5-FU and *P. g*-LPS (Fig. 1M). These results suggest that *P. g*-LPS inhibits 5-FU-induced cytotoxicity via TLR4 in OM-1 cells.

The impact of *E. coli*-LPS on 5-FU-induced cytotoxicity was examined in OM-1 cells and HOC621 cells. First, the cytotoxic effect of *E. coli*-LPS on OM-1 cells and HOC621 cells was examined. No significant change of LDH leakage was found in the presence of *E. coli*-LPS at concentrations of 1.0 µg/ml compared with the findings in the control (Fig. S2A and S2B). *E. coli*-LPS was thus employed at 1.0 µg/ml in the following experiments. 5-FU-induced leakage of LDH from damaged cells was significantly inhibited by *E. coli*-LPS in OM-1 cells and HOC621 cells (Fig.S2C and S2D). Additionally, the inhibition of LDH leakage by *E. coli*-LPS was alleviated by TAK-242 in 5-FU-treated OM-1 cells and HOC621 cells (Fig. S2E and S2F). These results suggest that TLR4 activation is involved in resistance to 5-FU-induced cytotoxicity in OM-1 cells and HOC621 cells.

### *P. g-*LPS-induced NF-κB activation is involved in resistance to 5-FU-induced cytotoxicity in OM-1 cells and HOC621 cells

**Fig. 2:**
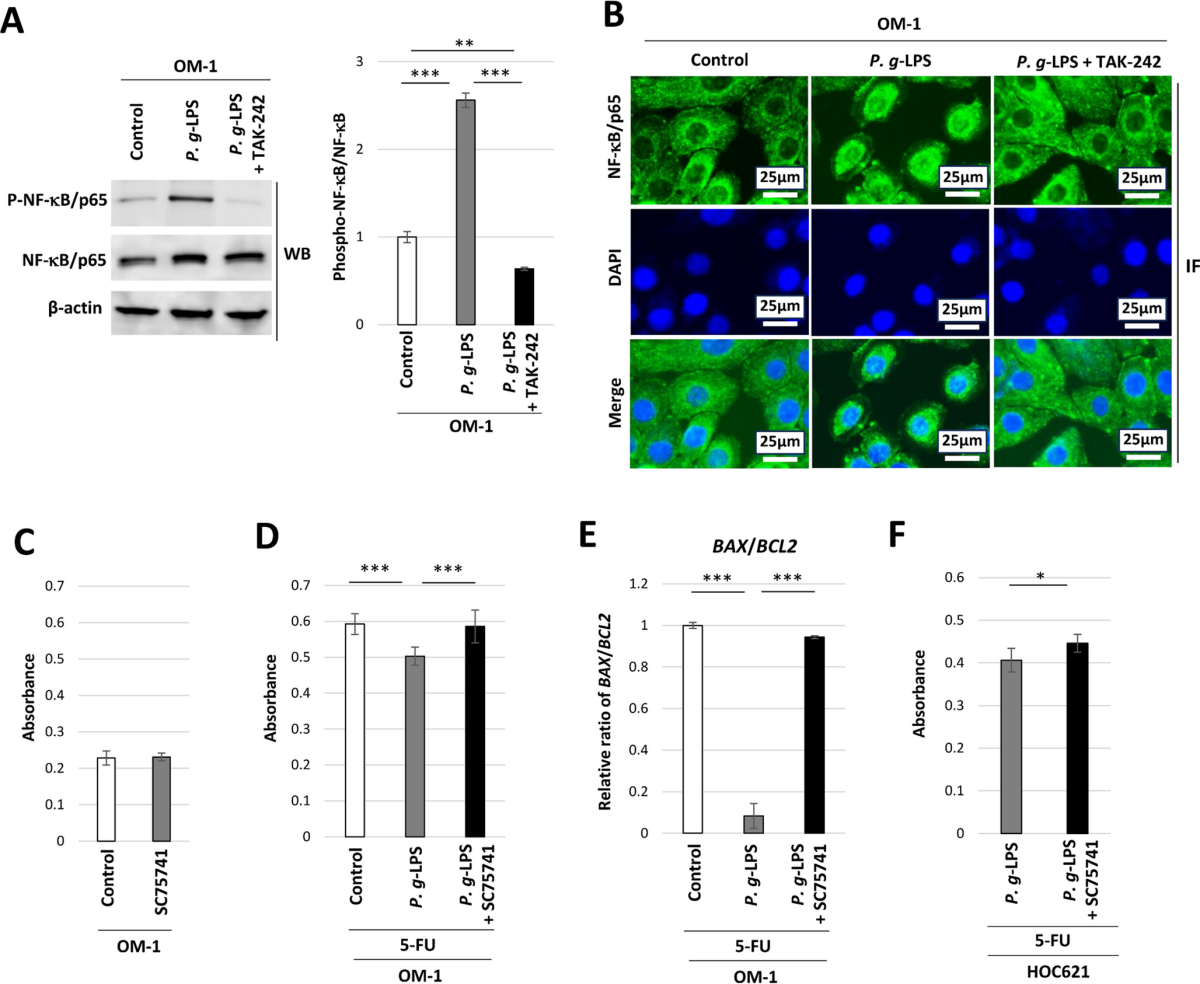
NF-κB activation in *P. g*-LPS-treated OM-1 cells. (**A**) Western blot showing the expression of NF-κB/p65 and phosphorylation of NF-κB/p65 in *P. g*-LPS-treated OM-1 cells and *P.g*-LPS + TAK-242-treated cells. The ratio of the band density of phospho-NF-κB/p65 protein to that of NF-κB/p65 (**P < 0.01, ***P < 0.001, one-way ANOVA with post-hoc Tukey’s HSD test). (**B**) Immunofluorescence staining of NF-κB/p65 (green) and nuclear counterstaining with 4′,6-diamidino-2-phenylindole (DAPI) (blue) in control OM-1 cells, *P. g*-LPS-treated cells, and *P. g*-LPS + TAK-242-treated cells. (**C**) Cytotoxicity assay in SC75741-treated OM-1 cells. (**D**) Cytotoxicity assay in *P. g*-LPS + SC75741-treated OM-1 cells in the presence of 5-FU (***P < 0.01, one-way ANOVA with post-hoc Tukey’s HSD test). (**E**) Relative expression ratio of *BAX*/*BCL2* mRNA in *P. g*-LPS + SC75741-treated OM-1 cells in the presence of 5-FU (***P < 0.01, one-way ANOVA with post-hoc Tukey’s HSD test). (**F**) Cytotoxicity assay in *P. g*-LPS + SC75741-treated HOC621 cells in the presence of 5-FU (*P < 0.05, unpaired t-test)

*P. g*-LPS-induced phosphorylation of NF-κB/p65 was inhibited by TAK-242 in OM-1 cells (Fig. 2A). The ratio of the band density of phospho-NF-κB/p65 protein to that of NF-κB/p65 was increased by *P. g*-LPS, but decreased by TAK-242 (Fig. 2A), in OM-1 cells. Nuclear staining of NF-κB/p65 was enhanced by *P. g*-LPS, which was inhibited by TAK-242, in OM-1 cells (Fig. 2B), indicating that the nuclear translocation of NF-κB/p65 (i.e., NF-κB activation) occurs in the presence of *P. g*-LPS. Next, the impact of the NF-κB inhibitor SC75741 on 5-FU-induced cytotoxicity was examined in OM-1 cells. No significant change in LDH leakage was found in the presence of SC75741 alone (Fig. 2C). *P. g*-LPS-inhibited LDH leakage was enhanced by SC75741 in the presence of 5-FU (Fig. 2D) in OM-1 cells. Additionally, the lowering of the relative mRNA expression ratio of *BAX*/*BCL2* by *P. g*-LPS was relieved by SC75741 treatment in the presence of 5-FU in OM-1 cells (Fig. 2E). Furthermore, *P. g*-LPS-inhibited LDH leakage was enhanced by SC75741 in the presence of 5-FU in HOC621 cells (Fig. 2F). These results suggest that *P. g*-LPS-induced NF-κB activation is involved in the resistance of OM-1 cells and HOC621 cells to 5-FU.

### *P. g*-LPS enhanced COX2 expression and facilitated PGE_2_ production in OM-1 cells

**Fig. 3:**
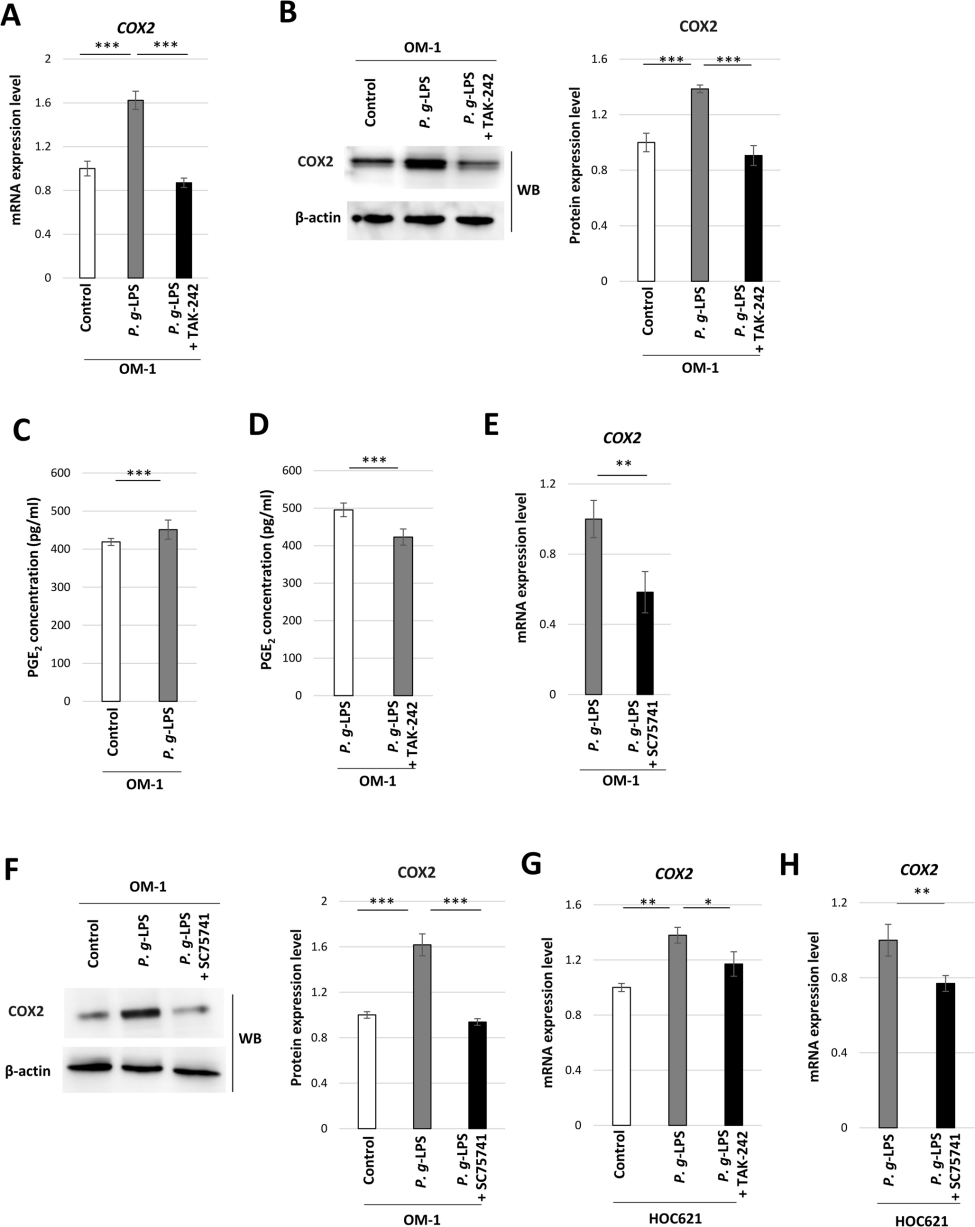
COX2 expression and PGE_2_ production in *P. g*-LPS-treated OM-1 cells. (**A**) *COX2* mRNA expression levels in *P. g*-LPS + TAK-242-treated OM-1 cells (***P < 0.001, one-way ANOVA with post-hoc Tukey’s HSD test). (**B**) Western blot showing COX2 protein expression in *P. g*-LPS + TAK-242-treated OM-1 cells. The ratio of the band density of COX2 protein to that of β-actin (***P < 0.001, one-way ANOVA with post-hoc Tukey’s HSD test). (**C**) PGE_2_ concentration of control OM-1 cells and *P. g*-LPS-treated cells (***P < 0.001, unpaired t-test). (**D**) PGE_2_ concentration of *P. g*-LPS + TAK-242-treated OM-1 cells (***P < 0.001, unpaired t-test). (**E**) *COX2* mRNA expression levels in *P. g*-LPS + SC75741-treated OM-1 cells (**P < 0.01, unpaired t-test). (**F**) Western blot showing COX2 protein expression in *P. g*-LPS + SC75741-treated OM-1 cells. The ratio of the band density of COX2 protein to that of β-actin (***P < 0.001, one-way ANOVA with post-hoc Tukey’s HSD test). (**G**) *COX2* mRNA expression levels in *P. g*-LPS + TAK-242-treated HOC621 cells (*P < 0.05, **P < 0.01, one-way ANOVA with post-hoc Tukey’s HSD test). (**H**) *COX2* mRNA expression levels in *P. g*-LPS + SC75741-treated HOC621 cells (**P < 0.01, unpaired t-test)

Next, the impact of *P. g*-LPS on COX2 expression was examined in OM-1 cells. *COX2* mRNA and COX2 protein expression was elevated by *P. g*-LPS (Fig. 3A and B), with these elevations being inhibited by the TLR4 inhibitor TAK-242 (Fig. 3A and B). Moreover, PGE_2_ concentration in culture medium was increased in the presence of *P. g*-LPS in OM-1 cells (Fig. 3C), which was downregulated by TAK-242 (Fig. 3D). Additionally, *COX2* mRNA and protein expression was inhibited by the NF-kB inhibitor SC75741 in the presence of *P. g*-LPS in OM-1 cells (Fig. 3E and F). These results suggest that *P. g*-LPS promotes the production of PGE_2_ by activating the TLR4–NF-κB–COX2 pathway in OM-1 cells.

*COX2* mRNA expression was enhanced by *P. g*-LPS in HOC621 cells, which was downregulated by TAK-242 (Fig. 3G). *COX2* mRNA expression was inhibited by SC75741 in the presence of *P. g*-LPS in HOC621 cells (Fig. 3 H). These results suggest that *P. g*-LPS attenuates 5-FU-induced cytotoxicity by activating the TLR4–NF-κB–COX2 pathway in HOC621 cells.

### *P. g*-LPS attenuated 5-FU-induced cytotoxicity by inducing PGE_2_ autocrine signaling in OM-1 cells

**Fig. 4:**
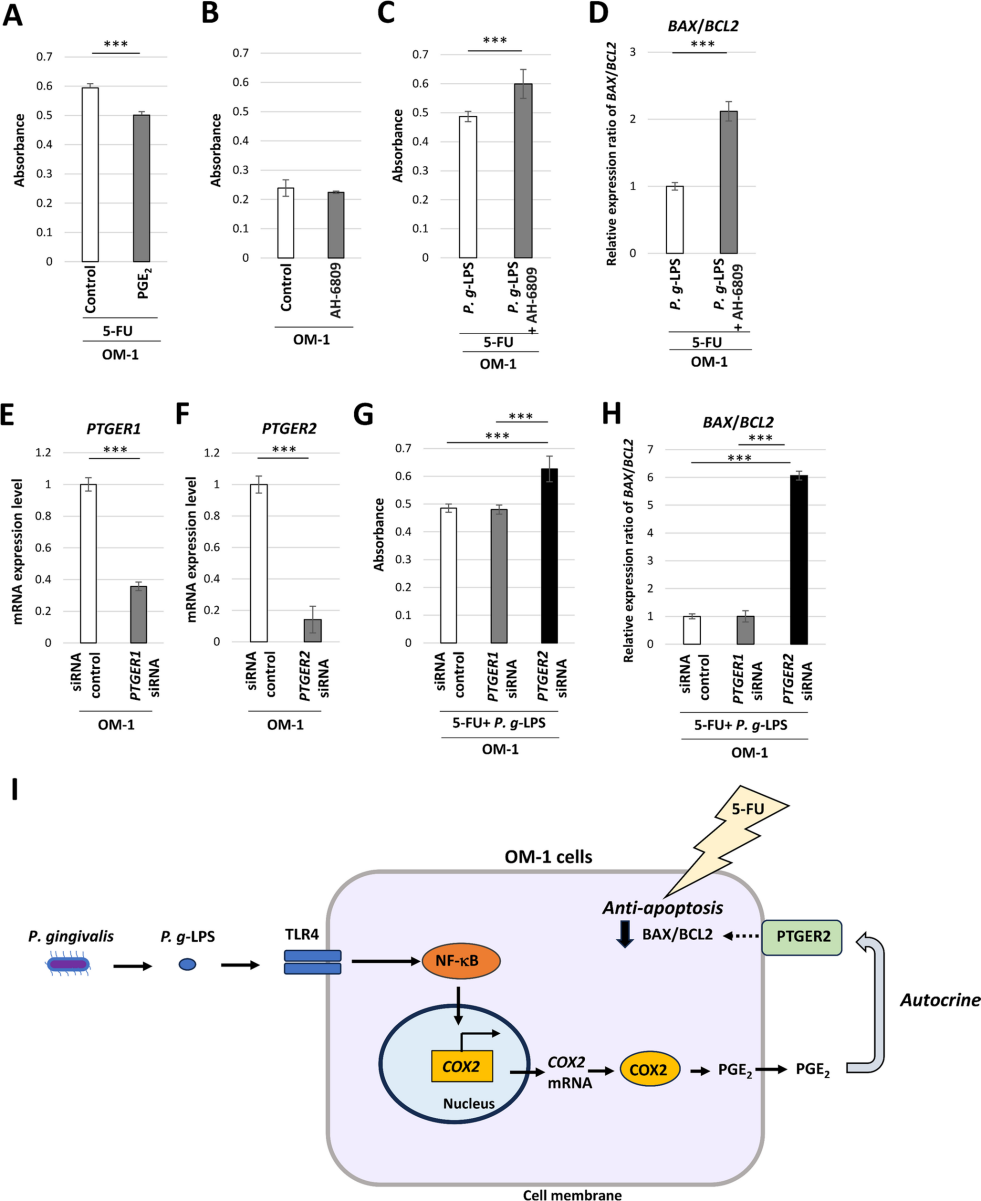
5-FU-induced cytotoxicity in *PTGER1* and *PTGER2* siRNA knockdown OM-1 cells in the presence of *P. g*-LPS. (**A**) Cytotoxicity assay in PGE_2_-treated OM-1 cells in the presence of 5-FU (***P < 0.001, unpaired t-test). (**B**) Cytotoxicity assay in AH-6809-treated OM-1 cells. (**C**) Cytotoxicity assay in *P. g*-LPS + AH-6809-treated OM-1 cells in the presence of 5-FU (***P < 0.001, unpaired t-test). (**D**) Relative expression ratio of *BAX*/*BCL2* mRNA in *P. g*-LPS + AH-6809-treated OM-1 cells in the presence of 5-FU (***P < 0.001, unpaired t-test). (**E**) *PTGER1* mRNA expression in *PTGER1* siRNA knockdown OM-1 cells (***P < 0.001, unpaired t-test). (**F**) *PTGER2* mRNA expression in *PTGER2* siRNA knockdown OM-1 cells (***P < 0.001, unpaired t-test). (**G**) Cytotoxicity assay in *PTGER1* and *PTGER2* siRNA knockdown OM-1 cells in the presence of 5-FU and *P. g*-LPS (***P < 0.001, one-way ANOVA with post-hoc Tukey’s HSD test). (**H**) Relative expression ratio of *BAX*/*BCL2* mRNA in *PTGER1* and *PTGER2* siRNA knockdown OM-1 cells in the presence of 5-FU + *P. g*-LPS (***P < 0.001, one-way ANOVA with post-hoc Tukey’s HSD test). (**I**) *P. g*-LPS activates the TLR4/NF-κB/COX2 pathway and enhances PGE_2_ production in OM-1 cells

5-FU-induced LDH leakage was inhibited by the addition of PGE_2_ (Fig. 4A), suggesting that exogenous PGE_2_ attenuates the cytotoxicity of 5-FU in OM-1 cells. Next, the impact of the prostaglandin E receptor 1 (PTGER1)*/*prostaglandin E receptor 2 (PTGER2) antagonist AH-6809 on *P. g*-LPS-induced resistance to 5-FU was examined in OM-1 cells. No significant increase in LDH leakage was found in AH-6809-treated cells compared with the level in untreated cells (Fig. 4B). Meanwhile, the inhibition of LDH leakage by *P. g*-LPS was relieved by AH-6809 in the presence of 5-FU in OM-1 cells. (Fig. 4C). The mRNA expression ratio of *BAX*/*BCL2* was also elevated by AH-6809 in the presence of 5-FU in OM-1 cells (Fig. 4D). These results suggest that endogenous PGE_2_ attenuates 5-FU-induced cytotoxicity via PTGER1/PTGER2 in OM-1 cells. The mRNA expression of *PTGER1* and *PTGER2* was significantly reduced in *PTGER1* and *PTGER2* siRNA knockdown cells, respectively (Fig. 4E and F). The inhibition of cytotoxicity by *P. g*-LPS was relieved by *PTGER2* siRNA knockdown in the presence of 5-FU in OM-1 cells (Fig. 4G), suggesting that PTGER2 is involved in resistance to 5-FU-induced cell death. The *P. g*-LPS-attenuated mRNA expression ratio of *BAX*/*BCL2* was also elevated after *PTGER2* siRNA knockdown in the presence of 5-FU in OM-1 cells (Fig. 4H). These results suggest that *P. g*-LPS inhibits 5-FU-induced cytotoxicity by enhancing PGE_2_ autocrine signaling in OM-1 cells (Fig. 4I).

### *P. g*-LPS induced migration and invasion of OM-1 cells

**Fig. 5:**
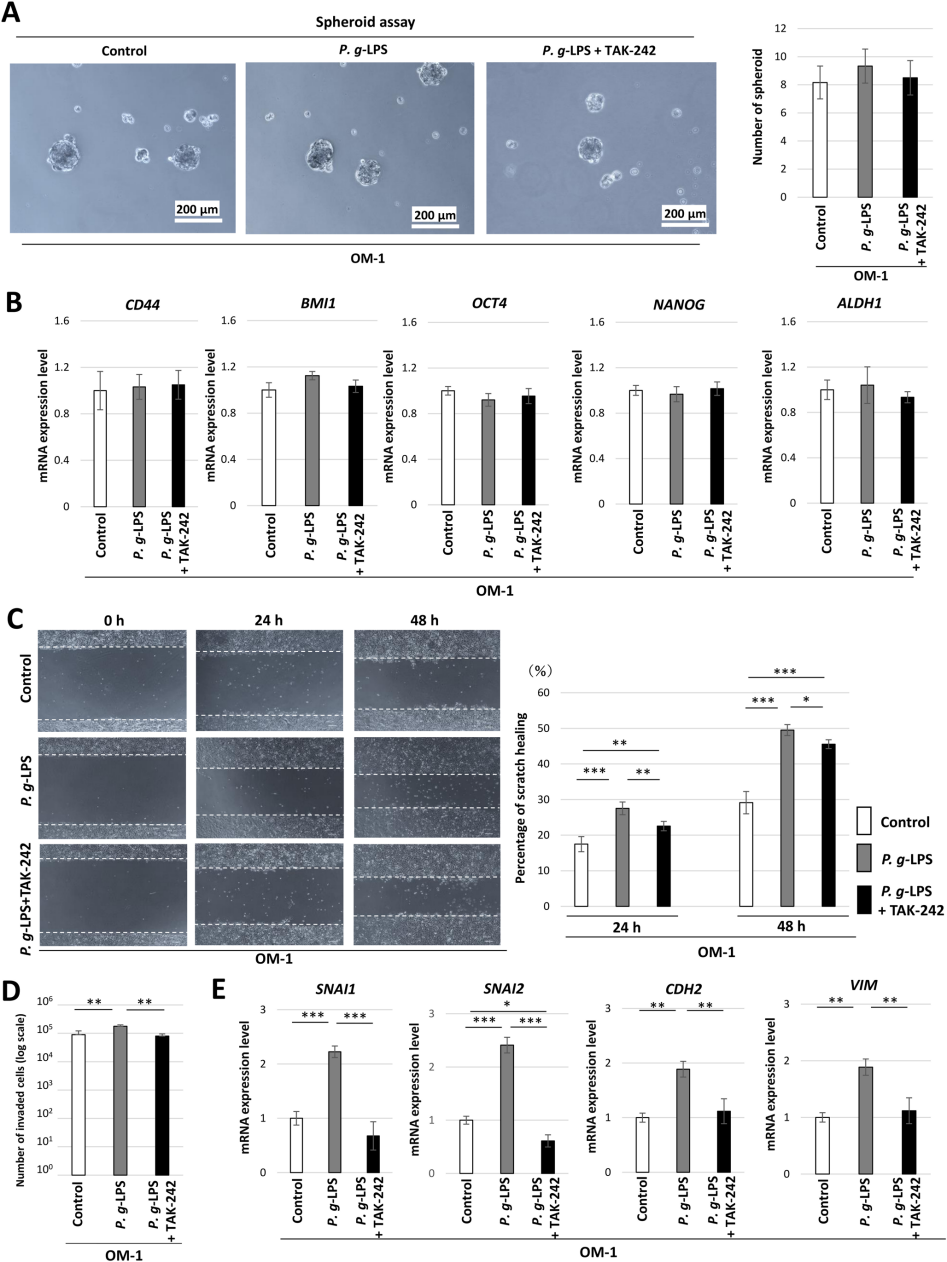
Tumor spheroid formation and cell migration ability in *P. g*-LPS-treated OM-1 cells. (**A**) Spheroid formation in control OM-1 cells, *P. g*-LPS-treated cells, and *P. g*-LPS + TAK-242-treated cells. The numbers of spheroids in control OM-1 cells, *P. g*-LPS-treated cells, and *P. g*-LPS + TAK-242- treated cells. (**B**) *CD44*, *BMI1*, *OCT4*, *NANOG*, and *ALDH1* mRNA expression levels in *P. g*-LPS + TAK-242-treated OM-1 cells. (**C**) Scratch assay at 0, 24, and 48 h in *P. g*-LPS-treated OM-1 cells, *P. g*-LPS + TAK-242-treated cells, and untreated cells. Proportion of scratch healing area in *P. g*-LPS-treated OM-1 cells, *P. g*-LPS + TAK-242-treated cells, and untreated cells at 24 and 48 h (*P < 0.05, **P < 0.01, ***P < 0.001, one-way ANOVA with post-hoc Tukey’s HSD test). (**D**) The number of invading cells among *P. g*-LPS-treated OM-1 cells, *P. g*-LPS + TAK-242-treated cells, and untreated cells (**P < 0.01, one-way ANOVA with post-hoc Tukey’s HSD test). (**E**) *SNAI1*, *SNAI2*, *CDH2*, and *VIM* mRNA expression levels in *P. g*-LPS + TAK-242-treated OM-1 cells (*P < 0.05, **P < 0.01, ***P < 0.001, one-way ANOVA with post-hoc Tukey’s HSD test)

Spheroid formation ability was examined in OM-1 cells and HOC621 cells using low-adherence culture plates (Fig. 5A, Fig. S3). There was no significant difference in the number of spheroids between *P. g*-LPS-treated cells and untreated cells in either OM-1 cells or HOC621 cells (Fig. 5A, Fig. S3). Additionally, the mRNA expression of cancer stem cell marker genes such as *CD44*, *BMI1*, *OCT4*, *NANOG*, and *ALDH1* was examined in OM-1 cells in the presence of *P. g*-LPS. No significant change in expression of cancer stem cell markers was found in the presence of *P. g*-LPS or both *P. g*-LPS and TAK-242 in OM-1 cells (Fig. 5B).

The results of the wound healing assay at 0, 24, and 48 h are shown in Fig. 5C. The proportion of scratch healing was greater in *P. g*-LPS-treated OM-1 cells than in untreated cells at 24 and 48 h, while this increase in healing was inhibited by the TLR4 inhibitor TAK-242 at both of these time points (Fig. 5C). The findings also showed that *P. g*-LPS increased the number of invading cells, with this increase being inhibited by TAK-242 in OM-1 cells (Fig. 5D). The mRNA expression of mesenchymal markers such as *SNAI1*, *SNAI2*, *CDH2*, and *VIM* was upregulated by *P. g*-LPS in OM-1 cells. In line with the findings above, these increases were in turn reduced by TAK-242 (Fig. 5E). Additionally, *P. g*-LPS-enhanced scratch healing and mRNA expression of mesenchymal markers were inhibited by SC75741 in OM-1 cells (Fig. S4A, S4B). The mRNA expression of mesenchymal markers such as *SNAI2*, *CDH2*, and *VIM* was upregulated by *P. g*-LPS in HOC621 cells (Fig S5A). However, there was no significant difference in the proportion of scratch healing between control HOC621 cells and *P. g*-LPS-treated cells at 24 and 48 h (Fig. S5B).

### *P. g*-LPS promoted cell protrusions and inhibited 5-FU-induced cytotoxicity in amoeboid CD44^high^ OM-1 cells

**Fig. 6:**
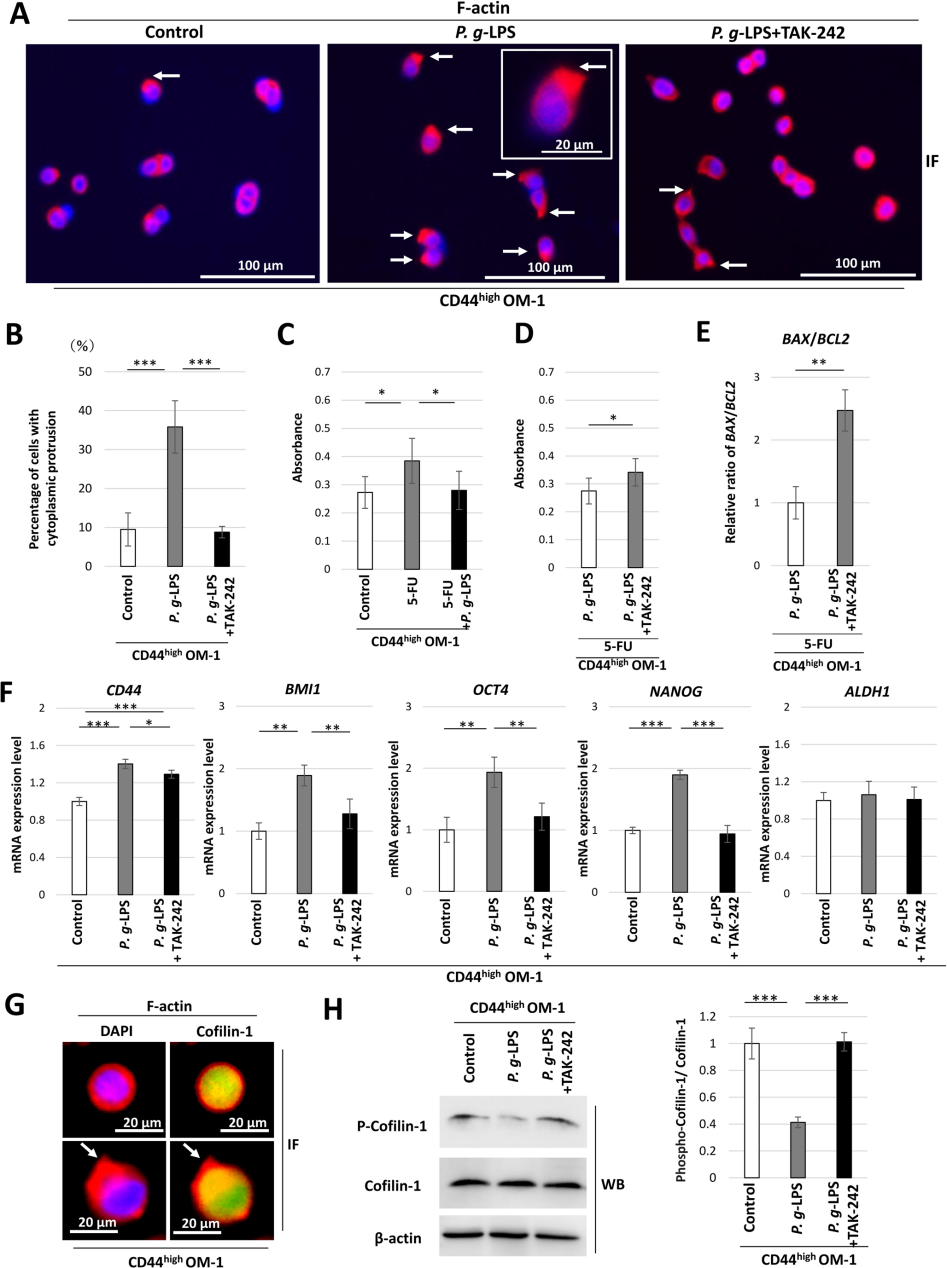
Cofilin-1 and cytoplasmic protrusions in *P. g*-LPS-treated CD44^high^ OM-1 cells. (**A**) Immunofluorescence of F-actin (red) and DAPI (blue) in control CD44^high^ OM-1 cells, *P. g*-LPS-treated cells, and *P. g*-LPS + TAK-242-treated cells. Cytoplasmic protrusion (arrow). (**B**) The proportion of amoeboid cells with cytoplasmic protrusions among control CD44^high^ OM-1 cells, *P. g*-LPS-treated cells, and *P. g*-LPS + TAK-242-treated cells (***P < 0.001, one-way ANOVA with post-hoc Tukey’s HSD test). (**C**) Cytotoxicity assay in *P. g*-LPS-treated CD44^high^ OM-1 cells in the presence of 5-FU (*P < 0.05, one-way ANOVA with post-hoc Tukey’s HSD test). (**D**) Cytotoxicity assay in *P. g*-LPS + TAK-242-treated CD44^high^ OM-1 cells in the presence of 5-FU (*P < 0.05, unpaired t-test). (**E**) Relative expression ratio of *BAX*/*BCL2* mRNA in *P. g*-LPS + TAK-242-treated CD44^high^ OM-1 cells in the presence of 5-FU (**P < 0.01, unpaired t-test). (**F**) *CD44*, *BMI1*, *OCT4*, *NANOG*, and *ALDH1* mRNA expression levels in *P. g*-LPS + TAK-242-treated CD44^high^ OM-1 cells (*P < 0.05, **P < 0.01, ***P < 0.001, one-way ANOVA with post-hoc Tukey’s HSD test). (**G**) Immunofluorescence of F-actin (red), Cofilin (green), and nuclear counterstaining with 4′,6-diamidino-2-phenylindole (DAPI) (blue). Cytoplasmic protrusion (arrow). (**H**) Western blot showing expression of Cofilin-1 and phosphorylated-Cofilin-1 in *P. g*-LPS-treated CD44^high^ OM-1 cells and *P. g*-LPS + TAK-242-treated cells. The ratio of the band density of phosphorylated-Cofilin-1 protein to that of Cofilin-1 (***P < 0.001, one-way ANOVA with post-hoc Tukey’s HSD test)

CD44^high^ OM-1 cells were cultured on silicone gel-coated culture plates to investigate the impact of *P. g*-LPS on amoeboid CD44^high^ cells with a cancer stem cell-like phenotype. Immunofluorescence of F-actin in amoeboid CD44^high^ OM-1 cells was shown irrespective of the presence or absence of *P. g*-LPS (Fig. 6A). Many amoeboid cells with actin-rich cytoplasmic protrusions were found in the presence of *P. g*-LPS (Fig. 6A). The number of such cells was counted in 10 fields per well using immunofluorescence images of F-actin under a fluorescence microscope and the proportion relative to the total number of counted cells was calculated. The findings showed that the proportion of cells with cytoplasmic protrusions was significantly increased in the presence of *P. g*-LPS (Fig. 6B). Meanwhile, these *P. g*-LPS-induced cytoplasmic protrusions were attenuated by the TLR4 inhibitor TAK-242 (Fig. 6B).

Next, the impact of *P. g*-LPS on 5-FU-induced cytotoxicity was examined in amoeboid CD44^high^ OM-1 cells. In line with the earlier findings, 5-FU-induced LDH leakage was inhibited in the presence of *P. g*-LPS (Fig. 6C), which was relieved by TAK-242 (Fig. 6D). Additionally, the *BAX*/*BCL2* ratio was significantly induced by TAK-242 in the presence of 5-FU and *P. g*-LPS (Fig. 6E). These results suggest that *P. g*-LPS attenuates 5-FU-induced cytotoxicity via TLR4 activation in amoeboid CD44^high^ OM-1 cells, as well as in their parental OM-1 cells. Furthermore, significant increases in *CD44*, *BMI1*, *OCT4*, and *NANOG* mRNA expression were found in the presence of *P. g*-LPS (Fig. 6F), which were inhibited by TAK-242 (Fig. 6F).

Cofilin-1 expression was also found in the amoeboid CD44^high^ OM-1 cells and cells with cytoplasmic protrusions (Fig. 6G). The dephosphorylation of Cofilin-1 (reflecting its activation) was induced by *P. g*-LPS, while TAK-242 recovered this by promoting Cofilin-1’s phosphorylation (Fig. 6H). These results suggest that *P. g*-LPS may be involved in cytoplasmic protrusions via Cofilin-1 activation in amoeboid CD44^high^ OM-1 cells.

## Discussion

Risk factors for oral cancer include smoking, alcohol consumption, inadequate consumption of fresh fruit and vegetables, and human papillomavirus (HPV) infection [28, 29]. Additionally, periodontopathic bacteria such as *Porphyromonas*, *Fusobacterium*, *Actinomyces*, and *Candida* species are known to be important in oral carcinogenesis [30]. Interestingly, the prevalence of HPV16 and herpesvirus, as well as periodontopathic bacteria, in the oral cavity was reported to be associated with periodontal inflammation in older people [31, 32]. It is assumed that changes in the composition of the oral microbiome, which is influenced by periodontitis, contribute to the development of oral cancer. Specifically, periodontopathic bacteria may be involved in oral carcinogenesis by conferring proliferative and anti-apoptotic abilities on oral epithelial cells by inducing long-term inflammation locally.

It has been reported that immunohistochemical analysis revealed a greater abundance of *P. gingivalis* in OSCC gingival tissue than in normal gingival tissue [33]. In addition, *P. gingivalis* was found to be more abundant in saliva specimens from OSCC patients than in specimens from individuals without OSCC [34]. In vitro analysis also revealed that *P. gingivalis* promotes pro-matrix metalloproteinase (MMP)9 activation and invasion in OSCC cells via activation of the MAPK/NF-κB pathway under co-coculture with *P. gingivalis* and OSCC cells [35]. Additionally, *P. gingivalis* was shown to facilitate the secretion of MMPs and IL-8 in OSCC cells [36]. These results suggest that *P. gingivalis* is a potential risk factor for the development of oral cancer and its metastasis.

Components of microorganisms in the oral cavity, such as peptidoglycans, lipoteichoic acids, and LPS, induce the production of proinflammatory cytokines [37], which results in continuous inflammation and systemic chronic inflammatory disease. LPS was also shown to enhance growth and migration via the induction of COX2 expression in breast cancer cells and non-small cell lung cancer cells [38, 39]. In addition, *P. g*-LPS was reported to enhance the migratory ability of glioma cells by activating AKT [40]. Elsewhere, in OSCC cells, *P. g*-LPS was found to promote cell viability and induce the expression of EMT-related genes [41]. In this study, *P. g*-LPS was found to enhance the migration and invasiveness of OM-1 cells. Therefore, the LPS-activated TLR4/ NF-κB pathway may be involved in the migratory ability of OSCC cells. However, no significant enhancement of migration was found in *P. g*-LPS-treated HOC621 cells. *P. g-*LPS-induced migratory ability may thus vary depending on the particular OSCC cell line. *P. g*-LPS also enhanced cofilin-1 activation in CD44^high^ OM-1 cells, suggesting that it plays a vital role in cytoplasmic protrusion-based migration in OSCC cancer stem cells. These are important findings given that cancer stem cells with the ability to self-renew are responsible for tumor maintenance and recurrence. Against this background, the results obtained here suggest that *P. g*-LPS may be involved in poor prognosis in OSCC patients.

In OSCC cells, Orphan nuclear receptor family 4 A was reported to be involved in 5-FU resistance in a manner dependent on PGE_2_ [11]. The autocrine signal PGE_2_ is involved in the induction of BCL2 via the PGE receptor in oral cancer cells, which results in anti-apoptotic effects [19]. Furthermore, in this study, *P. g*-LPS was shown to be involved in chemotherapy resistance by activating the TLR4–COX2 pathway and enhancing PGE_2_ autocrine mechanisms in OM-1 cells. These results highlight the importance of high levels of PGE_2_ in the tumor microenvironment for resistance to anti-cancer drugs in OSCC.

There are limitations in this in vitro study. We exposed OSCC cells to 250 µg/mL 5-FU in this work, in accordance with our previous study [11]. Such a concentration of 5-FU is higher than the 5-FU concentrations (2.0–50 µg/mL) typically used for in vitro chemotherapy response assays [42–44]. As such, we additionally confirmed that cell cytotoxicity induced by a lower concentration of 5-FU (25 µg/mL) was inhibited by *P. g*-LPS. However, the 5-FU concentration used for in vitro chemotherapy response assays may not reflect clinically relevant exposure because the plasma concentration of 5-FU was reported to be very low (i.e., 0.1–1.0 µg/mL) in oral cancer patients who had 5-FU therapy [45]. Therefore, it remains unclear whether *P. g*-LPS inhibits the cytotoxic effect of 5-FU in oral cancer patients. As a second limitation of this study, regarding the CD44^high^ subpopulation, this cell type comprises a large proportion of OM-1 cells, as we previously reported [18]. However, other OSCC cell lines in our laboratory showed small CD44^high^ subpopulations. Therefore, we could not obtain an adequate number of FACS-sorted CD44^high^ cells from other OSCC cell lines to immediately use for the subsequent experiments. *P. g-*LPS-induced chemotherapy resistance of the CD44^high^ subpopulation in other OSCC cell lines remains unknown.

## Conclusion

*P. g*-LPS induces chemotherapy resistance by activating the TLR4/NF-kB/COX2 pathway and enhancing PGE_2_ autocrine signaling in OM-1 cells. *P. g*-LPS may be involved in the acquisition of a highly malignant phenotype by OSCC cells.

## Supplementary Information

Below is the link to the electronic supplementary material.

Supplementary Material 1—Figure S1. (A) Cytotoxicity assay in 5-FU (25 μg/mL) + *P. g*-LPS-treated OM-1 cells (***P < 0.001, one-way ANOVA with post-hoc Tukey’s HSD test). (B) Relative expression ratio of *BAX*/*BCL2* mRNA in 5-FU (25 μg/mL) + *P. g*-LPS-treated OM-1 cells (**P < 0.01, ***P < 0.001, one-way ANOVA with post-hoc Tukey’s HSD test).

Supplementary Material 2—Figure S2. (A) Cytotoxicity assay in the presence of *E. coli*-LPS at concentrations of 1.0 μg/ml in OM-1 cells. (B) Cytotoxicity assay in the presence of *E. coli*-LPS at concentrations of 1.0 μg/ml in HOC621 cells. (C) Cytotoxicity assay in 5-FU + 1.0 μg/ml *E. coli*-LPS-treated OM-1 cells (***P < 0.001, one-way ANOVA with post-hoc Tukey’s HSD test). (D) Cytotoxicity assay in 5-FU + 1.0 μg/ml *E. coli*-LPS-treated HOC621 cells (*P < 0.05, ***P < 0.001, one-way ANOVA with post-hoc Tukey’s HSD test). (E) Cytotoxicity assay in *E. coli*-LPS + TAK-242-treated OM-1 cells in the presence of 5-FU (***P < 0.001, unpaired t-test). (F) Cytotoxicity assay in *E. coli*-LPS + TAK-242-treated HOC621 cells in the presence of 5-FU (**P < 0.01, unpaired t-test)

Supplementary Material 3—Figure S3. Spheroid formation in control HOC621 cells and *P. g*-LPS-treated cells. The numbers of spheroids in control HOC621 cells and *P. g*-LPS-treated cells.

Supplementary Material 4—Figure S4. (A) Scratch assay at 0, 24, and 48 h in* P. g*-LPS-treated OM-1 cells and *P. g*-LPS + SC75741-treated cells. Proportion of scratch healing area in *P. g*-LPS-treated OM-1 cells and *P. g*-LPS + SC75741-treated cells at 24 and 48 h (*P < 0.05, Tukey’s multiple comparison test). (B) *SNAI1*, *SNAI2*, *CDH2*, and *VIM* mRNA expression levels in* P. g*-LPS + SC75741-treated OM-1 cells (*P < 0.05, **P < 0.01, ***P < 0.001, unpaired t-test).

Supplementary Material 5—Figure S5. (A) *SNAI1*, *SNAI2*, *CDH2*, and *VIM* mRNA expression levels in control HOC621 cells and *P. g-*LPS-treated cells (***P < 0.001, unpaired t-test). (B) Scratch assay at 0, 24 and 48 h in control HOC621 cells and* P. g*-LPS-treated cells. Proportion of scratch healing area in control HOC621 cells and *P. g*-LPS-treated cells at 24 and 48 h.

Supplementary Material 6 (Table S1)

Supplementary Material 7 (Table S2)

Supplementary Material 8 (Table S3)

## Data Availability

All data generated or analyzed in this study are included in this paper.
