# Supplementary figures and images for: Porphyromonas gingivalis-derived lipopolysaccharide confers chemotherapy resistance and migratory ability on oral cancer cells by activating toll-like receptor 4 signaling pathway
Source: Mol Biol Rep. 2026 Mar 27;53(1):551. doi: 10.1007/s11033-026-11713-1 (PMC13031245; doi:10.1007/s11033-026-11713-1)

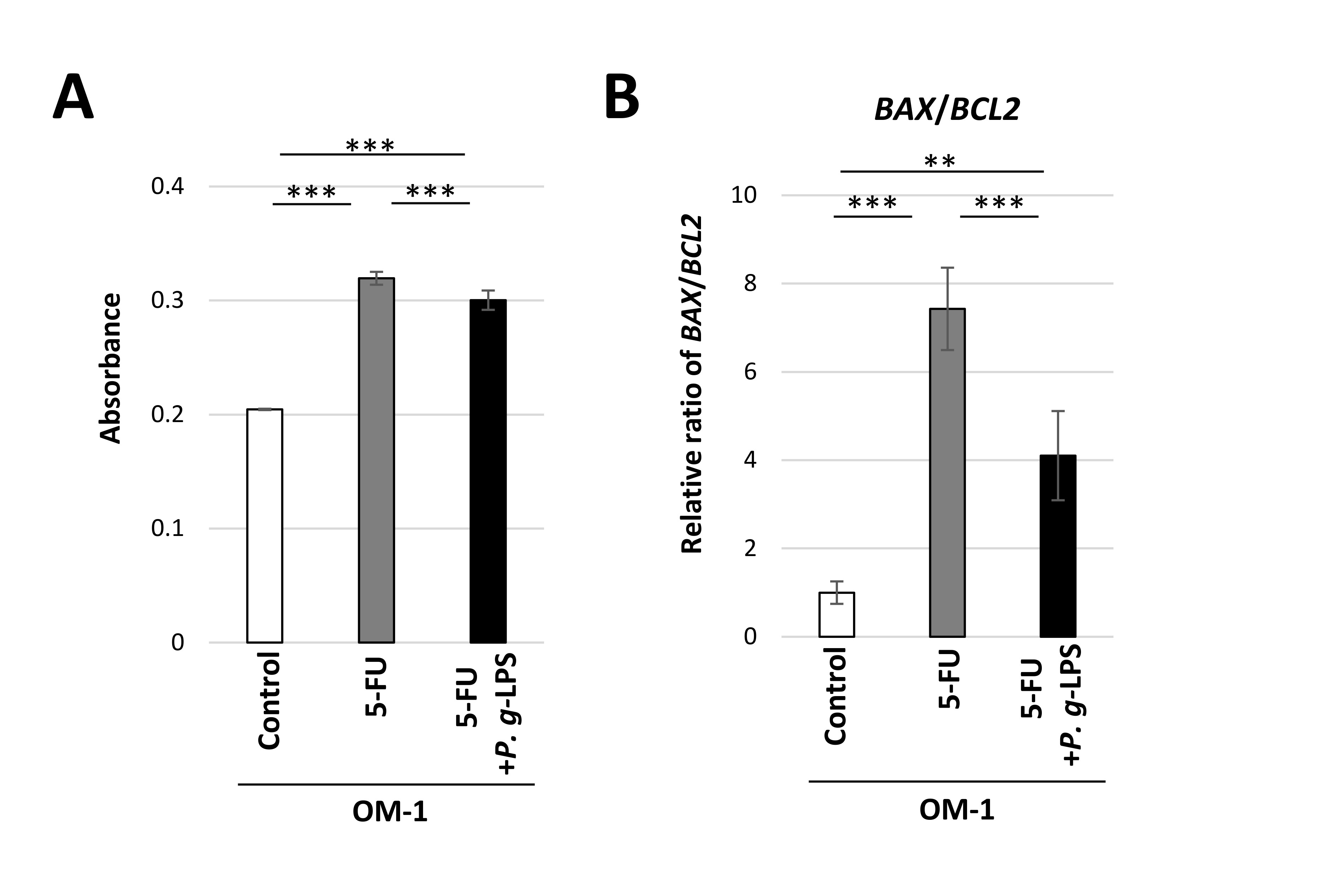

Supplement: Supplementary file 1 — Supplementary Material 1—Figure S1. (A) Cytotoxicity assay in 5-FU (25 μg/mL) + P. g-LPS-treated OM-1 cells (***P < 0.001, one-way ANOVA with post-hoc Tukey’s HSD test). (B) Relative expression ratio of BAX/BCL2 mRNA in 5-FU (25 μg/mL) + P. g-LPS-treated OM-1 cells (**P < 0.01, ***P < 0.001, one-way ANOVA with post-hoc Tukey’s HSD test). [file 11033_2026_11713_MOESM1_ESM.jpg]

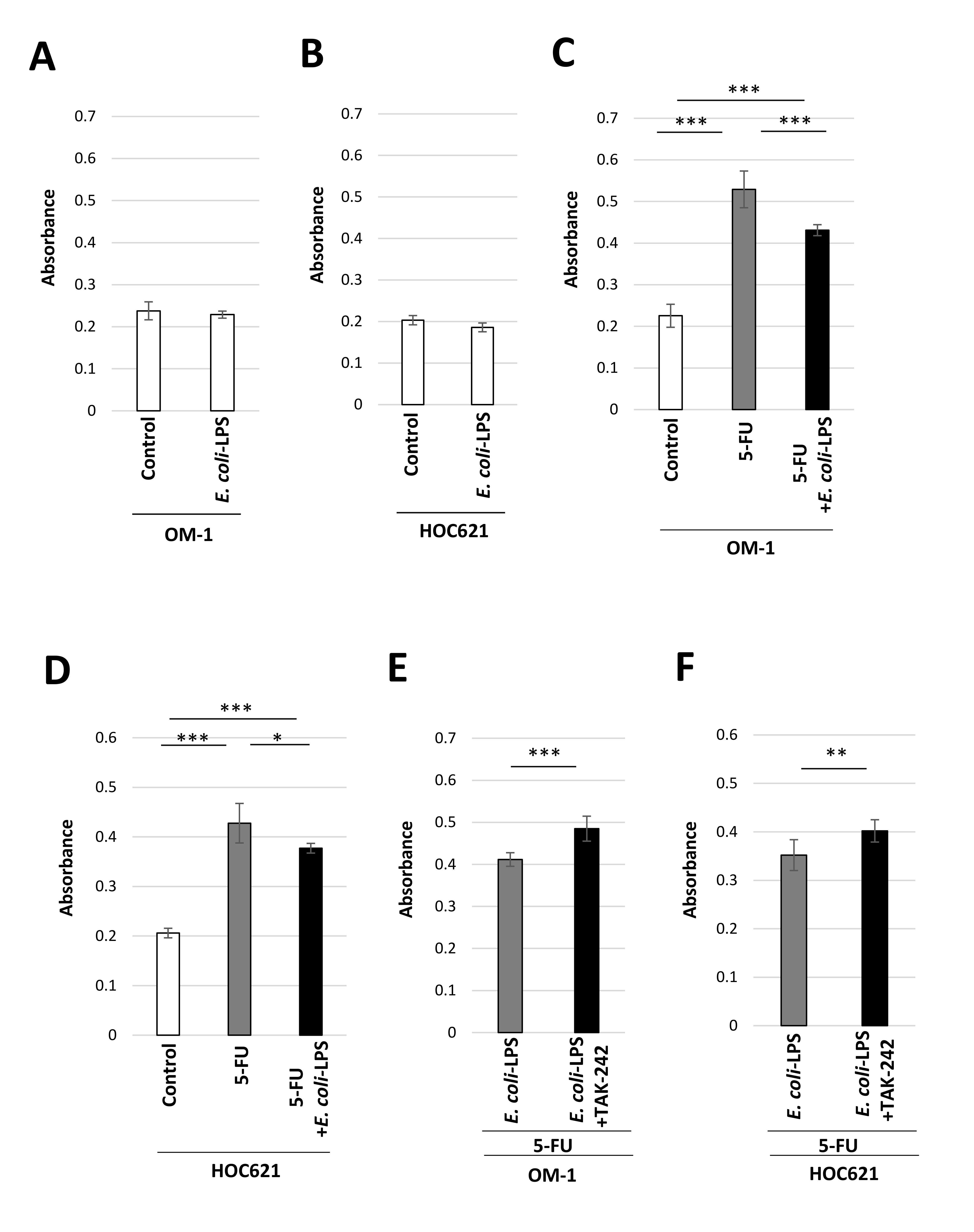

Supplement: Supplementary file 2 — Supplementary Material 2—Figure S2. (A) Cytotoxicity assay in the presence of E. coli-LPS at concentrations of 1.0 μg/ml in OM-1 cells. (B) Cytotoxicity assay in the presence of E. coli-LPS at concentrations of 1.0 μg/ml in HOC621 cells. (C) Cytotoxicity assay in 5-FU + 1.0 μg/ml E. coli-LPS-treated OM-1 cells (***P < 0.001, one-way ANOVA with post-hoc Tukey’s HSD test). (D) Cytotoxicity assay in 5-FU + 1.0 μg/ml E. coli-LPS-treated HOC621 cells (*P < 0.05, ***P < 0.001, one-way ANOVA with post-hoc Tukey’s HSD test). (E) Cytotoxicity assay in E. coli-LPS + TAK-242-treated OM-1 cells in the presence of 5-FU (***P < 0.001, unpaired t-test). (F) Cytotoxicity assay in E. coli-LPS + TAK-242-treated HOC621 cells in the presence of 5-FU (**P < 0.01, unpaired t-test) [file 11033_2026_11713_MOESM2_ESM.jpg]

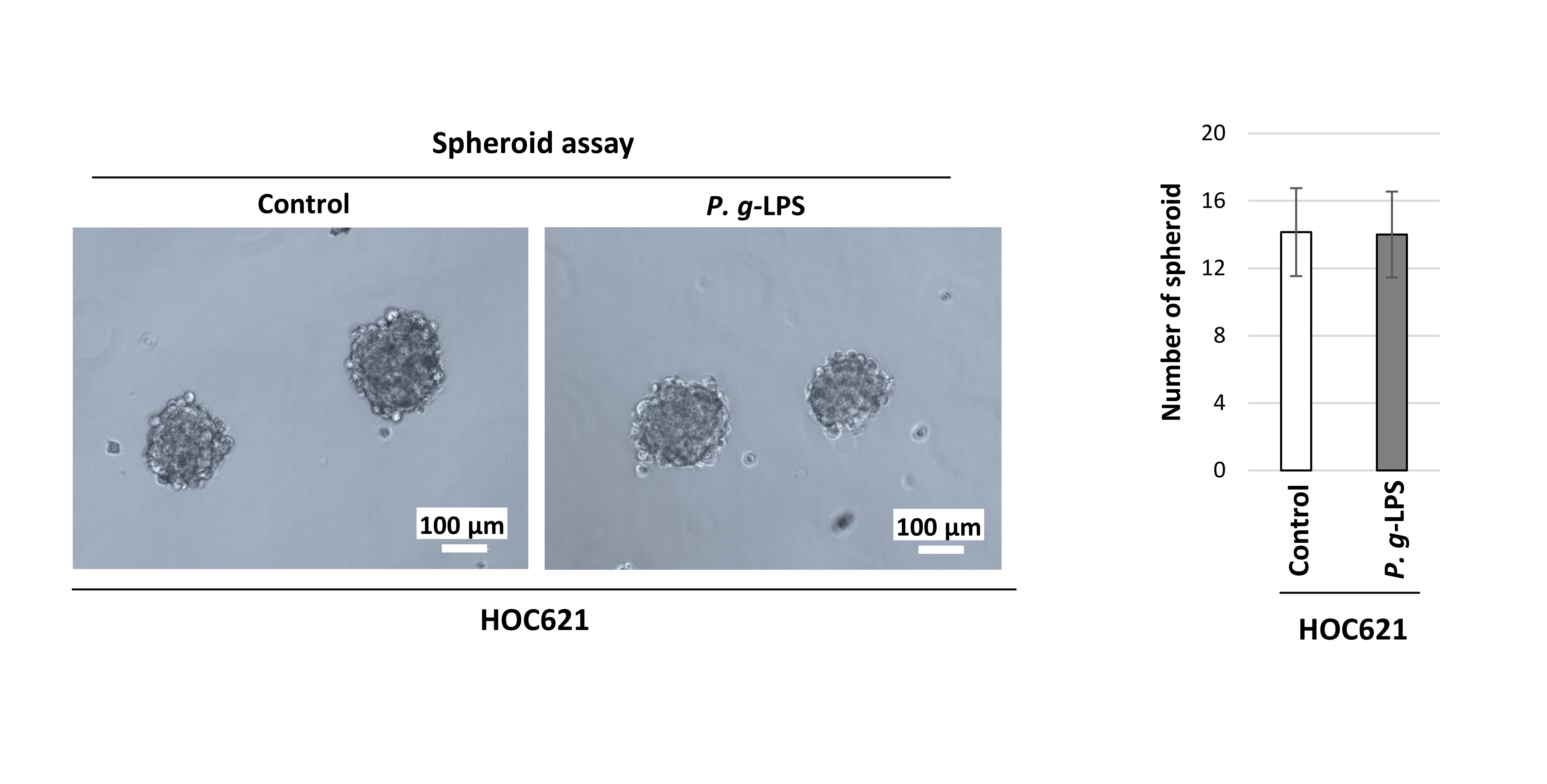

Supplement: Supplementary file 3 — Supplementary Material 3—Figure S3. Spheroid formation in control HOC621 cells and P. g-LPS-treated cells. The numbers of spheroids in control HOC621 cells and P. g-LPS-treated cells. [file 11033_2026_11713_MOESM3_ESM.jpg]

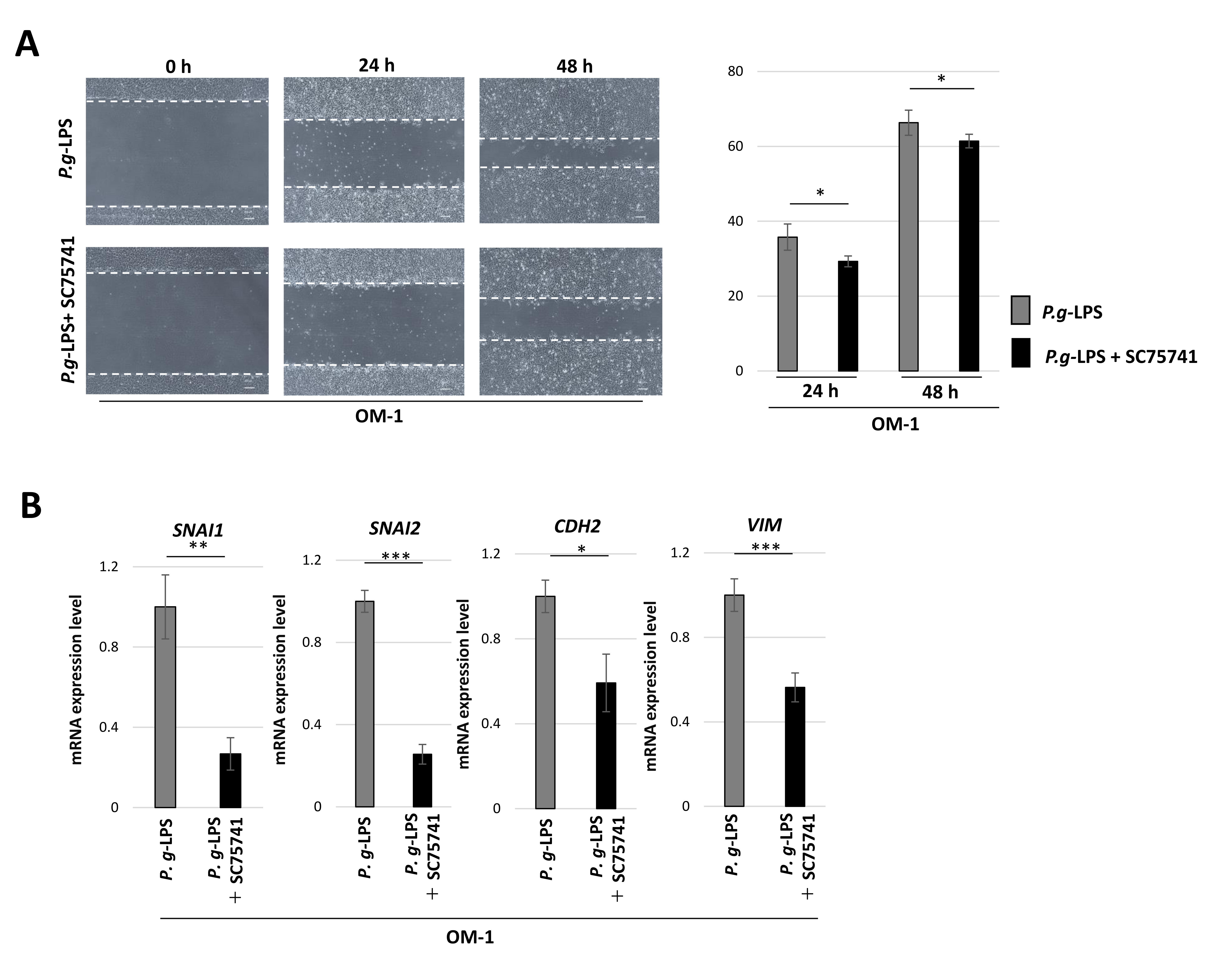

Supplement: Supplementary file 4 — Supplementary Material 4—Figure S4. (A) Scratch assay at 0, 24, and 48 h in P. g-LPS-treated OM-1 cells and P. g-LPS + SC75741-treated cells. Proportion of scratch healing area in P. g-LPS-treated OM-1 cells and P. g-LPS + SC75741-treated cells at 24 and 48 h (*P < 0.05, Tukey’s multiple comparison test). (B) SNAI1, SNAI2, CDH2, and VIM mRNA expression levels in P. g-LPS + SC75741-treated OM-1 cells (*P < 0.05, **P < 0.01, ***P < 0.001, unpaired t-test). [file 11033_2026_11713_MOESM4_ESM.jpg]

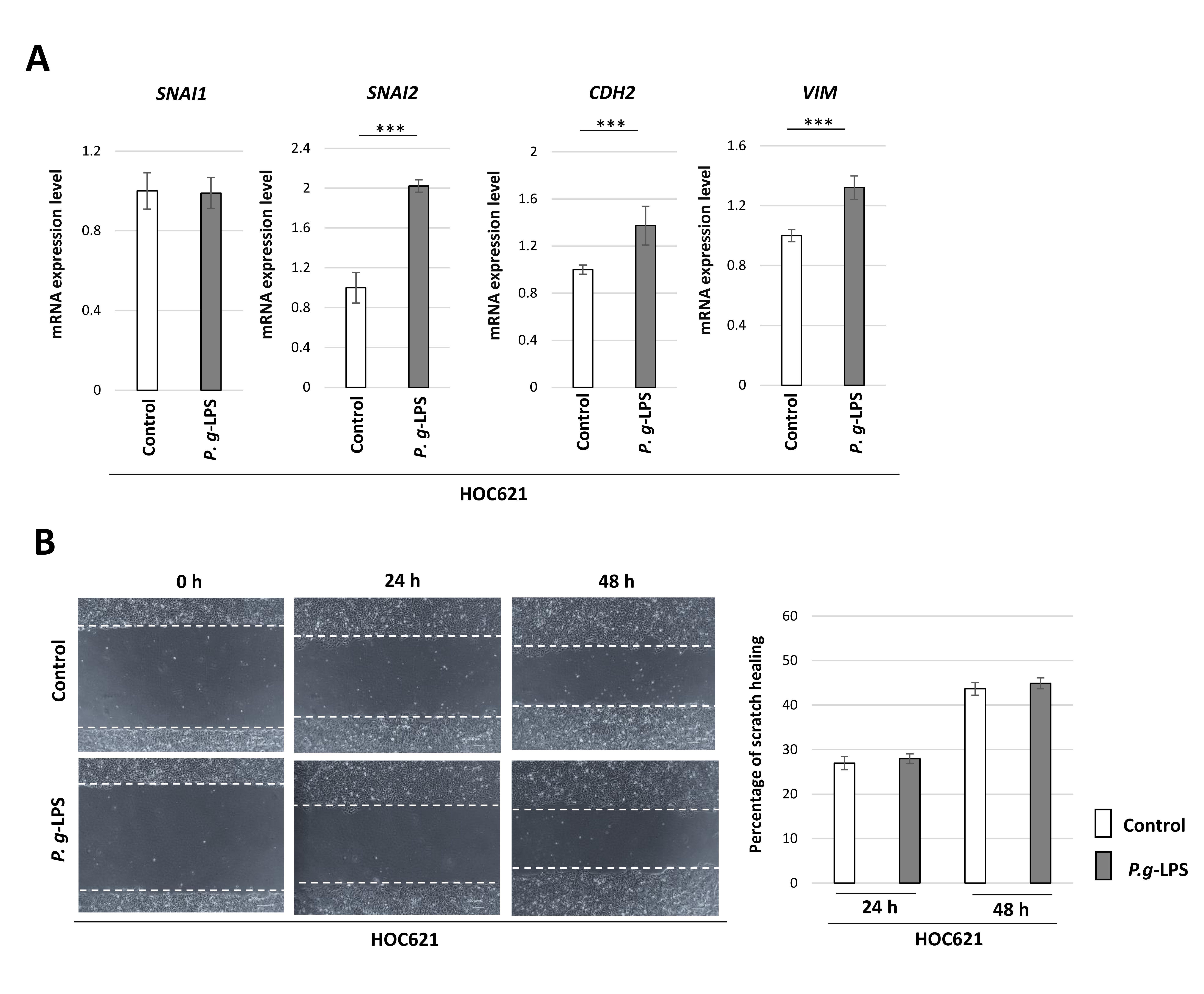

Supplement: Supplementary file 5 — Supplementary Material 5—Figure S5. (A) SNAI1, SNAI2, CDH2, and VIM mRNA expression levels in control HOC621 cells and P. g-LPS-treated cells (***P < 0.001, unpaired t-test). (B) Scratch assay at 0, 24 and 48 h in control HOC621 cells and P. g-LPS-treated cells. Proportion of scratch healing area in control HOC621 cells and P. g-LPS-treated cells at 24 and 48 h. [file 11033_2026_11713_MOESM5_ESM.jpg]
