# Supplementary material for: Porphyromonas gingivalis-derived lipopolysaccharide confers chemotherapy resistance and migratory ability on oral cancer cells by activating toll-like receptor 4 signaling pathway
Source: Mol Biol Rep. 2026 Mar 27;53(1):551. doi: 10.1007/s11033-026-11713-1 (PMC13031245; doi:10.1007/s11033-026-11713-1)
Supplement: Supplementary file 6 — Supplementary Material 6 (Table S1) [file 11033_2026_11713_MOESM6_ESM.docx]

**Table S1. The sequences of siRNA used in this study**

| **Gene** | **siRNA sequence** |
| --- | --- |
| *TLR4* | 5′-GAGCCUAAGCCACCUCUCUACCUUA**-**3′ |
|  | 5′**-**UAAGGUAGAGAGGUGGCUUAGGCUC-3′ |
| *PTGER1* | 5′-AGAUGCACGACACCACCAUGAUACC**-**3′ |
|  | 5′**-**GGUAUCAUGGUGGUGUCGUGCAUCU-3′ |
| *PTGER2* | 5′-CCUGCAACUUCAGUGUCAUUCUCAA**-**3′ |
|  | 5′**-**UUGAGAAUGACACUGAAGUUGCAGG-3′ |
