# Supplementary material for: Porphyromonas gingivalis-derived lipopolysaccharide confers chemotherapy resistance and migratory ability on oral cancer cells by activating toll-like receptor 4 signaling pathway
Source: Mol Biol Rep. 2026 Mar 27;53(1):551. doi: 10.1007/s11033-026-11713-1 (PMC13031245; doi:10.1007/s11033-026-11713-1)
Supplement: Supplementary file 7 — Supplementary Material 7 (Table S2) [file 11033_2026_11713_MOESM7_ESM.docx]

**Table S2. The sequences of primers employed in this study**

| **Gene** | **Primer sequence** | **P** **Annealing temperature (˚C)** | **Reference no.** |
| --- | --- | --- | --- |
| *BAX* | Forward: 5′-GGCCCACCAGCTCTGAGCAGA-3′  Reverse: 5′-GCCACGTGGGCGTCCCAAAGT-3′ | 58 | 19 |
| *BCL2* | Forward: 5′-GGTGCCACCTGTGGTCCACCTG-3′  Reverse: 5′-CTTCACTTGTGGCCCAGATAGG-3′ | 60 | 20 |
| *TLR4* | Forward: 5′-ACCTGTCCCTGAACCCTAT-3′  Reverse: 5′-CTAAACCAGCCAGACCTTG-3′ | 60 | 21 |
| *COX2* | Forward: 5′-TTGCTGGAACATGGAATTACC-3′  Reverse: 5′-TGCCTGCTCTGGTCAATG-3′ | 58 | 22 |
| *PTGER1* | Forward: 5′-TTGTCGGTATCATGGTGGTG-3′  Reverse: 5′-CCTGGCGCAGTAGGATGTAC-3′ | 58 | 23 |
| *PTGER2* | Forward: 5′-ATTCTCCTGGCTATCATGAC-3′  Reverse: 5′-GAACAGGAGGCCTAAGGATG-3′ | 58 | 23 |
| *CD44* | Forward: 5′-TCCAACACCTCCCAGTATGACA-3′  Reverse: 5′-GGCAGGTCTGTGACTGATGTACA-3′ | 58 | 24 |
| *BMI1* | Forward: 5′-CGTGTATTGTTCGTTACCTGGAGAC-3′  Reverse: 5′-CATTGGCAGCATCAGCAGAAGG-3′ | 58 | 25 |
| *OCT4* | Forward: 5′-GATGGCGTACTGTGGGCCC-3′  Reverse: 5′-CAAAACCCGGAGGAGTCCCA-3′ | 60 | 18 |
| *NANOG* | Forward: 5′-CAAAGGCAAACAACCCACTT-3′  Reverse: 5′-ACCAGACCCAGAACATCCAG-3′ | 60 | 18 |
| *ALDH1* | Forward: 5′-TACTCACCGATTTGAAGATT-3′  Reverse: 5′-TTGTCAACATCCTCCTTATC-3′ | 60 | 18 |
| *SNAI1* | Forward: 5′-GCTGCAGGACTCTAATCCAGA-3′  Reverse: 5′-ATCTCCGGAGGTGGGATG′-3′ | 58 | 26 |
| *SNAI2* | Forward: 5′-GGGGAGAAGCCTTTTTCTTG-3′  Reverse: 5′-TCCTCATGTTTGTGCAGGAG-3′ | 58 | 26 |
| *CDH2* | Forward: 5′-GCCCCTCAAGTGTTACCTCAA-3′  Reverse: 5′-AGCCGAGTGATGGTCCAATTT-3′ | 58 | 27 |
| *VIM* | Forward: 5′-CCCTCACCTGTGAAGTGGAT-3′  Reverse: 5′-GACGAGCCATTTCCTCCTTC-3′ | 58 | 27 |
| *GAPDH* | Forward: 5′-GTGAACCATGAGAAGTATGACAAC-3′  Reverse: 5′-ATGAGTCCTTCCACGATACC-3′ | 58 | 27 |
