# Supplementary material for: Porphyromonas gingivalis-derived lipopolysaccharide confers chemotherapy resistance and migratory ability on oral cancer cells by activating toll-like receptor 4 signaling pathway
Source: Mol Biol Rep. 2026 Mar 27;53(1):551. doi: 10.1007/s11033-026-11713-1 (PMC13031245; doi:10.1007/s11033-026-11713-1)
Supplement: Supplementary file 8 — Supplementary Material 8 (Table S3) [file 11033_2026_11713_MOESM8_ESM.docx]

**Table S3. List of antibodies used for western blotting.**

| **Antibody** | **P** **Source** |
| --- | --- |
| NF-κB p65 rabbit monoclonal antibody | Cell Signaling Technology, Beverly, MA, USA |
| Phospho-NF-κB p65 (Ser536) rabbit monoclonal antibody | Cell Signaling Technology |
| COX2 rabbit monoclonal antibody | Cell Signaling Technology |
| Cofilin-1 rabbit monoclonal antibody | Cell Signaling Technology |
| Phospho-Cofilin-1 rabbit monoclonal antibody | Cell Signaling Technology |
| β-actin mouse monoclonal antibody | Proteintech, Rosemont, IL, USA |
| Horseradish peroxidase-conjugated goat anti-rabbit antibody | Proteintech |
| Horseradish peroxidase-conjugated goat anti-mouse antibody | Proteintech |
